# Syndrome de Ramsay Hunt

**DOI:** 10.11604/pamj.2015.22.171.8123

**Published:** 2015-10-21

**Authors:** Madiha Mahfoudhi, Rim Lahiani

**Affiliations:** 1 Service de Médecine Interne A, Hôpital Charles Nicolle, Tunis, Tunisie; 2 Service d'ORL, Hôpital Charles Nicolle, Tunis, Tunisie

**Keywords:** Syndrome de Ramsay Hunt, hypoacousie, virus varicelle zona, Ramsay Hunt Syndrome, hypoacusia, varicella zoster virus

## Image en medicine

Le syndrome de Ramsay-Hunt correspond à une réactivation du virus varicelle zona au niveau des cellules ganglionnaires sensitives du nerf facial chez des patients ayant présenté une primo-infection de varicelle. Il se manifeste par des lésions cutanées de la zone sensitive de Ramsay-Hunt, une paralysie faciale périphérique, et des signes audio-vestibulaires. Le pronostic est le plus souvent bon sous traitement anti-viral bien conduit. Patiente âgé de 65 ans, aux antécédents de varicelle, a consulté pour une fièvre, une otalgie droite et une éruption cutanée de l'oreille droite évoluant depuis 5 jours. A l'examen physique, il avait une hypoacousie, une éruption érythémateuse vésiculeuse au niveau de la zone de Ramsay Hunt de l'oreille droite et une paralysie faciale périphérique droite. L'examen audiométrique a révélé une discrète surdité de perception droite. L'IRM a objectivé une névrite du nerf facial droit. Le traitement s'est basé sur l'association aciclovir et corticoïdes. L’évolution était favorable avec amélioration partielle de la paralysie faciale.

**Figure 1:**
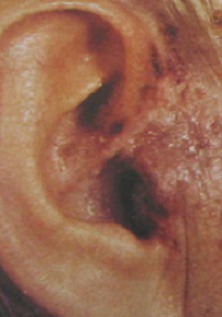
Erythème et vésicules cutanées de la zone de Ramsay Hunt droite

